# Prognostic Relevance of Pretherapeutic Gamma-Glutamyltransferase in Patients with Primary Metastatic Breast Cancer

**DOI:** 10.1371/journal.pone.0125317

**Published:** 2015-04-27

**Authors:** Christine Staudigl, Nicole Concin, Christoph Grimm, Georg Pfeiler, Regina Nehoda, Christian F. Singer, Stephan Polterauer

**Affiliations:** 1 Department of General Gynecology and Gynecologic Oncology, Comprehensive Cancer Center, Medical University of Vienna, Vienna, Austria; 2 Department of Obstetrics and Gynecology, Medical University of Innsbruck, Innsbruck, Austria; Wayne State University School of Medicine, UNITED STATES

## Abstract

**Background:**

Gamma-glutamyltransferase (GGT) is a known marker for apoptotic balance and cell detoxification. Recently, an association of baseline GGT levels and breast cancer incidence, tumor progression and chemotherapy resistance was shown. The purpose of this study was to evaluate the association of pre-therapeutic GGT levels, clinical-pathological parameters and survival in patients with primary metastatic breast cancer (PMBC).

**Methods:**

In this multicenter analysis, pre-therapeutic GGT levels and clinical-pathological parameters of 114 patients diagnosed with PMBC between 1996 and 2012 were evaluated. The association between GGT levels and clinical-pathological parameters were analysed. Patients were stratified into four GGT risk-groups (GGT < 18.00 U/L: normal low, 18.00 to 35.99 U/L: normal high, 36.00 to 71.99 U/L: elevated and ≥ 72.00 U/L: highly elevated) and survival analyses were performed.

**Findings:**

Patients in the high risk GGT group had a poorer overall survival, when compared to the low risk group with five-year overall survival rates of 39.5% and 53.7% (p = 0.04), respectively. Patients with larger breast tumors had a trend towards higher GGT levels (p = 0.053). Pre-therapeutic GGT levels were not associated with indicators of aggressive tumor biology such as HER2-status, triple negative histology, or poorly differentiated cancers.

**Conclusion:**

Pre-therapeutic GGT serum level might serve as a novel prognostic factor for overall-survival in patients with PMBC.

## Introduction

In Europe, breast cancer is the most frequently diagnosed malignancy in women with an incidence of 94 per 100 000 in 2012 [1]. Out of all breast cancer patients, 3–10% are diagnosed with metastatic breast cancer at initial diagnosis known as primary metastatic breast cancer (PMBC) [2, 3]. In this group of patients, median survival time ranges between 16–24 months and is influenced by prognostic factors like number and site of metastatic lesions as well as tumor characteristics such as hormonal receptor status and human epidermal growth factor 2-receptor (HER2) over-expression [4].

Gamma-glutamyltransferase (GGT) is a cell-membrane bound enzyme located in cells with high secretory activity like liver, kidneys and pancreas [5]. GGT is responsible for the glutathione (GSH) metabolism, catalyzing the degradation of extracellular GSH and further promoting amino-acid recovery for subsequent intracellular GSH synthesis [6]. Intracellular GSH acts as an antioxidant, neutralizing free radicals and so plays a decisive role in protection against oxidative stress during cell metabolism [6]. Therefore, GGT and GSH are increasing in circumstances of oxidative stress like carcinogenesis or cardiovascular diseases.

Serum GGT is a marker of liver dysfunction and excessive alcohol consumption [6]. GGT has been implicated in numerous other diseases like cardiovascular disease, diabetes, metabolic syndrome, malignancies and is associated with an increase in all-cause mortality [7–9]. Recently, a large quantitative review and two large epidemiologic cohort studies examined general cancer incidence and site specific cancer incidence in relation to GGT levels. They observed an association of increasing GGT levels and increased overall cancer risk as well as increased site specific risk for cancer in the breast-, male and female genital-organs, liver and digestive organs [10–12]. With respect to gynecologic cancers, elevated pre-therapeutic GGT levels were associated with worse prognosis in endometrial cancer and advanced tumor stage in cervical cancer [13, 14]. In ovarian cancer, increased GGT refers to both advanced tumor stage and worse survival [15]. Recent studies also investigated a possible association of elevated GGT levels and breast cancer but only found a positive correlation of elevated GGT and breast cancer incidence in premenopausal but not in postmenopausal women [16]. However, the large Swedish AMORIS study including 545 460 persons, identified elevated GGT levels as an independent risk-factor for breast cancer [17].

We investigated if GGT levels might be associated with poor prognosis and therefore useful as a novel prognostic marker in patients diagnosed with PMBC.

## Materials and Methods

### Patients

In the present multicenter study (Department of General Gynecology and Gynecologic Oncology, Comprehensive Cancer Center, Medical University of Vienna, Austria, n = 59 and Department of Obstetrics and Gynecology, Medical University Innsbruck, Tirol, Austria, n = 55) 114 consecutive patients with primary metastatic breast cancer diagnosed between the years 1996 and 2012 were included.

The study was approved by the Institutional review Commissions of the Ethics Committee: Medical University of Vienna (IRB approval number: 275/2009) and Medical University of Innsbruck (UN4144). There was no written informed consent obtained. Clinical pathological information was extracted retrospectively from the respective Breast Cancer Registries and by chart-review. Data was subsequently blinded and documented in a database. Only de-identified data was further analyzed.

We evaluated six different localizations of metastatic disease (lung/pleurae, liver, brain, bone, skin and other sites). The following data were evaluated: age at initial diagnosis, menopausal status, histological type, grading, hormonal receptor (HR) status (estrogen and progesterone receptor), HER2-status and tumor stage according to TNM-classification. Hormonal receptor positive cancer is defined as estrogen- and/or progesterone receptor positive.

Before initiating primary therapy, a laboratory work-up as well as a physical examination by an internal consultant was performed and results were documented. Patients who presented with pre-existing co-morbidities, which are known to be related to elevated GGT-levels other than metastases in the liver (i.e. alcohol abuse, pancreatic, and heart disease) were not included in the analysis.

### Clinical Management

Each patient had received standard staging procedures including chest x-ray or/and computer tomography (CT) of lungs and liver sonography or/and CT of liver as well as a bone scan. In dependence of these staging results and clinical symptoms, further measures like magnetic resonance tomography (MRT) and / or biopsy were performed to confirm metastatic disease.

Patients were treated according to the current available guidelines for PMBC disease [3, 18]. Hence, in dependence of performance status, co-morbidities, type and count of metastatic sites, tumor biology, hormonal- and HER2-status patients received either primary surgery of the tumor, systemic chemotherapy, endocrine therapy, palliative radiation therapy, targeted therapy and / or bone- directed agents [3].

### GGT Measurement

As part of the routine pre-treatment examination, blood samples for evaluation of serum GGT levels were obtained by peripheral venous puncture 24–48 h prior to start of therapy. GGT was determined in order to assess hepatic damage prior to treatment initiation. GGT concentrations were analyzed with an enzyme kinetic assay (Modular Hitachi 747 and Hitachi 917, Roche Diagnostics), as described elsewhere [5].

### Statistical Analysis

Variables are described by mean (standard deviation, SD) when normally distributed and by median (range) when skewed distributed (GGT). Student’s t-tests and One-Way ANOVA analyses were used to assess the association between pre-treatment serum GGT levels and clinical-pathological parameters where appropriate. Survival probabilities were calculated by the product limit method of Kaplan and Meier. Differences between groups were measured using the log-rank test. The results were analyzed for the endpoint of overall survival. Survival times of patients still alive or dead as a result of other causes than cancer were censored with the last follow-up date. A multivariate Cox regression model for overall survival was performed, comprising GGT risk groups (D and C vs. B and A), age (> 61 years vs. ≤ 61 years), T-stage (T3 and T4 vs. T2 vs. T1), histological grade (G3 vs. G2 and G1), hormonal receptor status (negative vs. positive), HER2 status (HER2 positive vs. HER2 negative), Triple negative cancer (HER2 and HR negative vs.HER2 or HR positive), metastases (others vs. bone) and menopausal status (postmenopausal vs. premenopausal). Patients were assigned to the previously established four GGT cancer risk groups [14, 15, 19] as follows: GGT < 18.00 U/L: group A (normal low), 18.00 to 35.99 U/L: group B (normal high), 36.00 to 71.99 U/L: group C (elevated) and ≥ 72.00 U/L: group D (highly elevated). *P*-values of <0.05 were considered statistically significant. Statistical software SPSS 18.0 for Mac (SPSS 18.0, SPSS Inc, Chicago, IL, USA) and SAS and SPlus (Version 2000 Professional, Redmond, WA, USA) were used for statistical analysis.

## Results

### Patients’ Characteristics

Detailed patients and tumor characteristics of the study cohort are presented in Table 1. Median (range) pre-therapeutic serum GGT level was 25.0 (7.0–514) U/L. After stratifying patients into the previously described GGT risk groups, 29 (25.4%) patients are assigned to group A (normal low GGT), 44 (38.6%) to group B (normal high GGT), 24 (21.1%) to group C (elevated GGT) and 17 (14.9%) to group D (highly elevated GGT) [13, 19].

**Table 1:** Characteristics of 114 patients with primary metastatic breast cancer.

Parameter	N or mean (SD)
**Total number of patients enrolled**	114
**Age at first diagnosis (years)**	61.0 (13.4)
Premenopausal	27 (23.7%)
Postmenopausal	83 (72.8%)
Unknown	4 (3.5%)
**Tumor histology, % of patients**	
Ductal invasive	82 (71.9%)
Lobular invasive	23 (20.2%)
Unknown	9 (7.9%)
**Tumor grade, % of patients**	
Well differentiated	1 (0.9%)
Moderate differentiated	61(53.5%)
Undifferentiated	40 (35.1%)
Unknown	12 (10.5%)
**T stage, % of patients**	
pT1	22 (19.3%)
pT2	18 (15.8%)
pT3	3 (2.6%)
pT4	37 (32.5%)
Unknown	34 (29.9%)
**N stage, % of patients**	
N0	16 (14.0%)
N1	13 (11.4%)
N2	15 (13.2%)
N3	12 (10.5%)
Unknown	58 (50.9%)
**Hormone receptor status, % of patients**	
HR positive	87 (76.3%)
HR negative	27 (23.7%)
**HER2 receptor status, % of patients**	
HER2 positive	17 (14.9%)
HER2 negative	94 (82.5%)
Unknown	3 (2.6%)
**Metastatic sites, % of patients**	
Bone	79 (69.3%)
Liver	37 (32.5%)
Lung	40 (35.1%)
Brain	17 (14.9%)
Skin	10 (8.8%)
Other locations	13 (11.4%)
**Follow-up**	
Median follow-up time, months	25.0 (27.7)
No. of deaths within follow-up period	48 (42.1%)

HR = hormonal receptor (estrogen and/or progesterone receptor), GGT = gamma-glutamyltransferase, HER2 = human epidermal growth factor 2-receptor

### Clinical-pathological Parameters

We found a trend towards an association of breast tumor-size and elevated pre-therapeutic GGT levels without reaching statistical significance (*p* = 0.053). GGT serum levels were not associated with patient’s age, lymph node involvement, histological type, histological grade, or hormonal-receptor- and HER2-status. Furthermore, we found no association of GGT serum levels with site of metastatic disease (isolated bone metastases *vs*. others, hepatic metastases *vs*. others). All GGT serum levels of patients with PMBC broken down by clinical-pathological parameters are given in Table 2.

**Table 2:** Pre-therapeutic GGT serum levels of patients with primary metastatic breast cancer broken down by clinical-pathological parameters.

Parameter	Mean (SD) GGT serum levels (units/L)^⇞^	p-Value
**Age**		0.9
≤61 Years	46.6 (58.4)	
>61 Years	47.8 (77.2)	
**T stage**		0.053
T1 and T2	30.2 (20.9)	
T3 and T4	59.9 (93.6)	
**Histological Type**		0.7
IDC	46.4 (67.4)	
ILC	53.7 (80.0)	
**Lymph Node Involvement** ^+^		0.2
Negative	42.1 (56.1)	
Positive	29.5 (20.7)	
**Histological Grade** *		0.7
G1	13.0 (N/A)	
G2	40.8 (42.7)	
G3	49.7 (86.8)	
**Hormonal Receptor**		
Estrogen receptor		0.54
- Negative	40.8 (47.7)	
- Positive	49.6 (74.1)	
Progesterone receptor		0.3
- Negative	40.2 (43.2)	
- Positive	52.7 (82.1)	
Hormonal receptor		0.3
- Negative	36.1 (31.5)	
- Positive	50.7 (75.5)	
**HER2-status**		0.9
Negative	48.3 (71.2)	
Positive	46.1 (53.7)	
**Triple negative Cancers**		0.5
Negative	49.8 (73.9)	
Positive	39.5 (34.7)	

* One-way ANOVA

^+^ Student’s T-test

^⇞^ Values are given as mean (standard deviation), GGT = gamma-glutamyltransferase, HER2 = human epidermal growth factor 2-receptor, SD = standard deviation

### Survival Analyses

**Fig 1:**
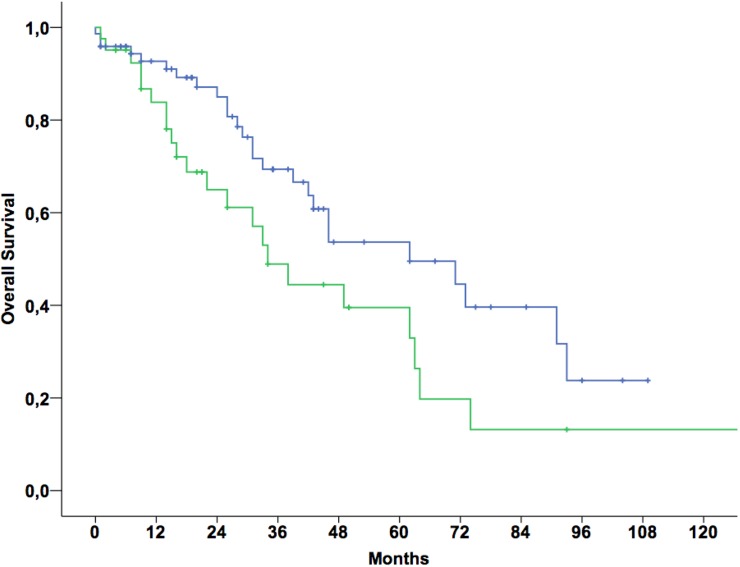
Kaplan-Meier curves for pre-therapeutic GGT levels groups A and B (n = 73, upper line) vs. C and D (n = 41, lower line) and overall survival (*P-value* for log-rank test <0.041).

High risk GGT groups were associated with poor overall survival in univariate analysis. Results of univariate and multivariable survival analyses are provided in Table 3. Fig 1 shows Kaplan-Meier curves for patients stratified to the low risk group (A and B) and to the high risk group (C and D) which had 5-year overall survival rates of 53.7% and 39.5%, respectively (Log-rank test, *p* = 0.041). The association of elevated GGT and survival could be confirmed in multivariable survival analyses (Table 3). Furthermore, in multivariable analyses poorly differentiated tumors, triple negative tumors and negative HR-status were associated with poor overall survival.

**Table 3:** Univariate and multivariable overall-survival analyses.

Parameter	5-year OS rate	Univariate^a^	Multivariable^b^
		HR (95% CI)	*P*-Value	HR (95% CI)	*P-*Value
**GGT risk groups**					
A and B (reference)	53.7%				
C and D	39.5%	1.8 (1.0–3.2)	0.04	4.1 (1.4–11.9)	0.01
Age*					
≤61 Years (reference)	49.1%				
>61 Years	47.2%	1.1 (0.6–1.9)	0.65	1.2 (0.5–3.2)	0.65
**T-Stage**					
T1 (reference)	65.5%				
T2	47.1%	0.5 (0.2–1.2)	0.13	0.3 (0.1–0.9)	0.027
T3 and T4	50.6%	0.8 (0.3–2.0)	0.68	0.4 (1.4–1.6)	0.23
**Grading**					
G1and G2 (reference)	60.2%				
G3	41.0%	1.4 (0.8–2.6)	0.29	5.4 (1.7–17.3)	0.004
**Hormonal Receptor Status**					
Positive (reference)	48.6%				
Negative	49.8%	0.7 (0.4–1.4)	0.33	0.03 (0.0–0.4)	0.013
**HER2 Status**					
Positive	27.7%	1.7 (0.8–3.4)	0.16	0.1 (0.0–1.1)	0.074
Negative (reference)	53.3%				
**Triple negative**					
HER2 or HR positive (reference)	45.9%				
HER2 and HR negative	60.2%	0.9 (0.5–2.0)	0.90	73.2 (4.1–1304.5)	0.003
**Metastases**					
Bone (reference)	44.7%				
Others	50.3%	0.7 (0.3–1.3)	0.22	1.6 (0.6–4.8)	0.38
**Menopausal Status**					
Premenopausal (reference)	57.3%				
Postmenopausal	47.2%	1.4 (0.7–2.8)	0.40	4.3 (0.7–27.0)	0.12

^a^Kaplan—Meier analysis (log-rank test)

^**b**^Multivariable Cox-regression analysis

*mean (SD) age: 61.0 (13.4) years; GGT = gamma-glutamyltransferase, HER2 = human epidermal growth factor 2-receptor, OS = overall survival, HR = hazard ratio, 95% CI = 95% Confidence Interval

## Discussion

In the present multi-centre study, we demonstrate that high pre-therapeutic serum GGT levels are significantly associated with decreased overall-survival in patients with PMBC. There is growing evidence that GGT is deregulated in malignant cells, and is involved in tumor progression towards more aggressive phenotypes associated with a worse prognosis by producing reactive oxygen species [20]. In terms of carcinogenesis, GGT clearly is a marker of oxidative stress. Nevertheless, it remains unclear whether GGT has a direct effect on or is an indicator of collateral damage [21]. If future studies show that GGT expression influences the biological behavior of breast cancer, GGT might even be an interesting novel target for anticancer therapeutics.

We stratified our patients into four well established risk groups according to their pre-therapeutic GGT serum levels [13]. Patients in the high risk groups C and D (elevated and highly elevated GGT) had significantly decreased overall-survival times compared to patients in the low risk groups A and B (normal and normal high GGT) in univariate analysis. This correlates well with findings in other gynecologic cancer entities such as cervical-, endometrial- and ovarian cancer, as well as other metastatic cancers like metastatic colorectal cancer and renal cell carcinoma [13–15, 17, 22, 23]. Multivariable analyses also showed that GGT is besides the established prognostic factors like tumor size, grading, and hormonal receptor status an independent prognostic factor in patients with PMBC. If our findings are validated in large independent cohorts the prognostic groups might be used to stratify patients within clinical trials. Furthermore, in multivariable analyses poorly differentiated tumors, triple negative tumors and negative HR-status were associated with poor overall survival. However, because of the lack of significance in univariate analyes these results should be interpreted with caution. The majority of PMBC patients are incurable and the fundamental treatment goal is palliation with maintenance the quality of life. Therefore, each treatment decision has to be individually with respect to tumor burden, tumor biology, count and site of metastatic disease and performance status.

We evaluated a possible association between GGT and clinical-pathological parameters such as histological grade, hormonal receptor status and lymph node involvement. We observed a (non-significant) trend towards an association of larger tumor-size and high GGT serum levels. Patients with T1 and T2 tumors showed slightly lower mean (SD) GGT levels when compared to T3 and T4 tumors (30.2 [20.9] vs. 59.9 [93.6]). This observation seems plausible, because GGT is an established marker for oxidative imbalance, and protects cells against free radical injury. As a consequence GGT is often expressed in malignant tumors and larger tumors may be associated with an increased appearance of GGT expression—reflected by higher serum levels—when compared to smaller tumors [6]. Nevertheless, due to the lack of statistical significance these results need to be interpreted carefully and validation in future studies is warranted. Other clinical-pathological parameters were not associated with elevated pre-therapeutic GGT serum levels.

Interestingly, GGT serum levels were not elevated in patients with liver metastases when compared to other metastatic sites. Even when patients with bone metastases only were compared to patients with visceral metastases no difference could be found. This finding is consistent with findings of two studies on renal cell carcinoma, which indicated that GGT is a sensitive marker for metastatic disease but not specific for the metastatic site [23, 24]. However, it remains unclear if GGT itself plays a substantial role in neoplastic transformation of cells or it is impairing proliferative / apoptotic balance and thus influences tumor formation and progression [25].

Our study has several potential limitations. Due to its retrospective study design, for a few patients the data set was incomplete. For some patients only limited information about medication, which might also influence the GGT level, was available. Nonetheless, all patients had pre-therapeutic laboratory assessment, medical history, and were examined by an Internal consultant before treatment started. Thus, patients with clinically relevant diseases (alcohol abuse, hepatobiliary-diseases), which might affect GGT levels, were excluded of the study. As the study follow-up duration was about sixteen years, we considered that potential changes in survival to improvements in management might bias our results. Nevertheless, for a rare disease like primary metastatic breast cancer the number of patients (n = 114) is relatively high. Despite the potential limitations, our results are clinically meaningful and might be hypothesis generating for future trials.

The present study indicates, that pre-therapeutic GGT serum level might be a novel prognostic factor in patients with PMBC. Further scientific research is necessary to answer the question, whether GGT itself plays a role in neoplastic transformation or is a result of tumor progression and metastasis.
